# Structure and IR Spectra of 3(5)-Aminopyrazoles and UV-Induced Tautomerization in Argon Matrix

**DOI:** 10.3390/molecules26144299

**Published:** 2021-07-15

**Authors:** Alina Secrieru, Susy Lopes, Maria L. S. Cristiano, Rui Fausto

**Affiliations:** 1 CCMAR and Department of Chemistry and Pharmacy, FCT, Campus de Gambelas, University of Algarve, 8005-039 Faro, Portugal; asecrieru@ualg.pt; 2 Department of Chemistry, University of Liverpool, Liverpool L69 7ZD, UK; mcristi@ualg.pt; 3 CQC-IMS, Department of Chemistry, University of Coimbra, 3004-535 Coimbra, Portugal; rfausto@ci.uc.pt

**Keywords:** 3(5)-aminopyrazoles, UV-induced phototautomerism, matrix isolation, infrared spectroscopy, DFT and TD-DFT calculations, anharmonic frequencies

## Abstract

The prototropic tautomerism in 3(5)-aminopyrazoles was investigated by matrix isolation infrared (IR) spectroscopy, supported by DFT(B3LYP)/6-311++G(d,p) calculations. In consonance with the experimental data, the calculations predict tautomer 3-aminopyrazole (3AP) to be more stable than the 5-aminopyrazole (5AP) tautomer (calculated energy difference: 10.7 kJ mol^−1^; Gibbs free energy difference: 9.8 kJ mol^−1^). The obtained matrix isolation IR spectra (in both argon and xenon matrices) were interpreted, and the observed bands were assigned to the tautomeric forms with help of vibrational calculations carried out at both harmonic and anharmonic levels. The matrix-isolated compound (in argon matrix) was then subjected to in situ broadband UV irradiation (λ > 235 nm), and the UV-induced transformations were followed by IR spectroscopy. Phototautomerization of the 3AP tautomer into the 5AP form was observed as the strongly prevalent reaction.

## 1. Introduction

Like other nitrogen-containing heterocyclic compounds, pyrazoles (five-membered heterocycles with two adjacent nitrogen atoms) have been attracting a growing interest due to their unique features and extensive range of applications [1,2,3,4]. Pyrazoles are prone to prototopic tautomerism, which appears as a key factor in determining their chemical reactivity. Indeed, in these compounds, 1,2-H shifts between the two vicinal nitrogen atoms are in general easy, energetically accessible processes that lead to tautomerism. This tautomerism correlates structurally, for example, the identically-substituted 3- and 5-pyrazoles (from here after abbreviated as 3(5)-pyrazoles).

The 3(5)-aminopyrazoles are currently used as starting materials in many important chemical reactions. Their use spans from the synthesis of different types of compounds with applications in pharmaceutics and agrochemical industries to the development of functional materials [5,6,7,8]. In spite of their widespread practical interest, these compounds have only been scarcely studied from the structural point of view at the molecular level, and the available information about their vibrational properties and photochemistry is also lacking. This is particularly critical in the case of the non-substituted 3(5)-aminopyrazoles, for which, to the best of our knowledge, no previous experimental studies have been reported hitherto in the gas phase (or for the isolated molecules in general), while the theoretically available data are also very limited. Previously reported experimental investigations on 3(5)-aminopyrazoles have been carried out for a few substituted compounds in solution or in the solid state, either by NMR spectroscopy or X-ray diffraction [9,10,11,12], while past theoretical studies on 3(5)-aminopyrazoles focused mostly on the evaluation of the relative stabilities of the 3-amino vs. the 5-amino tautomer in the gas phase or in solution [13,14,15,16]. The last studies indicated that the 3-amino tautomer is more stable than the 5-amino tautomer, but the relative stability of pyrazole tautomers have been found to be very much dependent on the nature of the substituents [13,14,15,16].

In line with our previous investigations on the structure and reactivity of 3(5)-substituted pyrazoles, with focus on the 3(5)-amino analogue and the prototropic tautomerism phenomenon as a fundamental modulatory feature of the reactivity of the class [17], in the present work, we studied the tautomerism in unsubstituted 3(5)-aminopyrazoles using low-temperature matrix isolation IR spectroscopy (in argon and xenon matrices), combined with theoretical calculations performed at the density functional theory level with the B3LYP functional and the standard 6-311++G(d,p) split-valence triple-ζ basis set augmented with diffuse functions. Theoretical calculations were used to obtain structural information on the tautomeric species and on their spectroscopic properties in order to support the analysis of the experimentally obtained infrared spectroscopy data. In addition, the effect of in situ broadband UV (λ > 235 nm) irradiation of the matrix-isolated 3(5)-aminopyrazoles was investigated. As described below in detail, it was found that, under these experimental conditions, the dominant photoprocess observed in both argon and xenon matrices was the phototautomerization of 3AP into its 5AP tautomer. The present study aimed to contribute to a better understanding of the molecular properties of pyrazoles, in particular their vibrational properties and photo-induced intramolecular tautomerism.

## 2. Results and Discussion

### 2.1. Geometries and Energies

**Figure 1:**
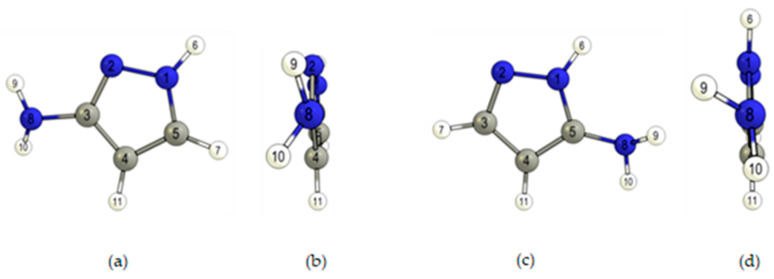
DFT(B3LYP)/6-311++G(d,p) optimized structures of 3AP (structures (**a**,**b**)) and 5AP (structures (**c**,**d**)) tautomers, with the atom numbering adopted in this work. Colors: C-grey, H-white and N-blue.

The 3(5)-aminopyrazoles considered in this study correspond to a pyrazole ring bearing an amine substituent at the position 3 or 5, respectively, in the case of 3-aminopyrazole (1*H*-pyrazol-3-amine, 3AP) and 5-aminopyrazole (1*H*-pyrazol-5-amine, 5AP) (Figure 1). These two tautomers can undergo interconversion via 1,2-*H* intramolecular shift and/or through intermolecular exchanges with neighboring molecules [17]. They are essentially rigid molecules with a single conformer each (in each case corresponding to a 4-fold symmetry degenerated structure). Table 1 shows the relevant structural parameters for the two tautomers.

The 3AP tautomer has its amine group with the hydrogen atoms making dihedral angles N_2_–C_3_–N_8_–H_9_ and N_2_–C_3_–N_8_–H_10_ equal to 15.1 and 143.6°, respectively, while the N_1_–C_5_–N_8_–H_9_ and N_1_–C_5_–N_8_–H_10_ dihedrals in 5AP are equal to 59.2 and −174.8°, i.e., in 3AP the amine H atom located closest to the plane of the ring (H_9_) stays 15.1° out of the plane, pointing towards the bare N_2_ atom, while in 5AP the amine hydrogen atom located closest to the plane of the ring (H_10_) is only 5.2° out of the ring plane and points towards C_4_. These structural differences are easy to rationalize: in 3AP, the positively charged H_9_ atom (+0.248 *e*; Mülliken charge) approaches the negatively charged undecorated N_2_ atom (−0.191 *e*), allowing also the second amine hydrogen atom (H_10_) to reduce the repulsive interaction with the ring H_11_ atom (+0.157 *e*); on the other hand, in 5AP both amine hydrogen atoms face a ring hydrogen atom, since in this case N_1_ is protonated, and the adopted geometry allows minimizing the repulsion with the ring H atom of larger charge, i.e., H_6_ (+0.297 *e*), bound to N_1_, compared with H_11_ (bound to C_4_), which has a charge of only +0.142 *e* (note that Mülliken populational analysis provides charge values that are very much dependent of the basis set and method used; however, even if the absolute values of the Mülliken atomic charges are basis set and method dependent, differences of the charge values calculated for structurally similar compounds are generally well predicted, at least qualitatively, so that we can expect that the observed trends are reliable). This geometry also allows the 5AP molecule to direct the amine group lone electron pair to a direction close to the ring N_1_–H_6_ bond, while in 3AP the lone pair is perpendicular to the ring (which favors electron delocalization through the π-system). Very interestingly, the tilt of the N_8_ amine nitrogen atom out of the plane of the ring assumes an identical value in the two tautomers (3.4°), while the degree of pyramidalization of N_8_ is somewhat more pronounced in 5AP compared with 3AP, as reflected in the relative values of the H_9_–N_8_–H_10_ angle (109.9° in 5AP, compared with 111.5° in 3AP), and of the C_5_–N_8_–(H_9_)–H_10_ improper dihedral angle in 5AP (127.3°) vs. the C_3_–N_8_–(H_9_)–H_10_ angle in 3AP (130.0°). The smaller degree of pyramidalization in 3AP (i.e., a more planar amine group) can also be correlated with a greater π electron delocalization between the amine group and the pyrazole ring in this tautomer, which is consistent with the different orientations adopted by the amine group in the two tautomers pointed out above and is also in agreement with the shorter C_3_–N_8_ bond length in 3AP (1.395 Å) compared with the C_5_–N_8_ bond length in 5AP (1.399 Å). As it can be seen in Table 1, the remaining related structural parameters do not differ very much in the two molecules.

The relative energy of the tautomers, as estimated by the DFT(B3LYP)/6-311++G(d,p) calculations, amounts to 10.7 kJ mol^−1^ in favor of 5AP, when zero-point vibrational energy contributions are taken into account, while the Gibbs energy difference between the two tautomers amounts to 9.8 kJ mol^−1^. This result follows the general trend previously noticed for pyrazoles substituted with π-electron donating groups [13,14,15,16]. Marín-Luna et al. [13] conducted a DFT(B3LYP) theoretical study on the basicity of a series of 150 pyrazole derivatives in both the gas phase and in aqueous solution and, for the 3(5)-aminopyrazoles studied in this work, their results fully agree with ours, the energy difference between the two tautomers reported by those authors matching the one presented in our work. Catalán et al. [14] carried out a semi-empirical INDO investigation for estimation of pK_a_ values in a series of azoles, also concluding that the 3AP tautomer should be more stable than the 5AP form, while Jarónczyk et al. [15] investigated the substituent effects on the pyrazole ring on the stability of the related tautomers at the MP2/6-311++G(d,p) level and concluded that electron donating groups such as Cl, F, OH and NH_2_ stabilize the structure that for the 3(5)-amino-substituted pyrazole corresponds to the 3AP tautomer, whereas electron withdrawing groups such as CFO, COOH and BH_2_ favor the tautomer structurally related to 5AP.

The reasons for the higher stability of the 3AP tautomer compared with 5AP were briefly addressed in Reference [15], through analysis of π and δ orbital interactions. The authors noticed the structural differences related with the orientation adopted by the amine group in the two tautomers, in particular the different orientation of their lone electron pair, stressing its importance for the stabilization of the 3AP form in consequence of an increased π-delocalization. As pointed out above, the present structural results fully confirm this hypothesis, while they also demonstrate the relevance of electrostatic interactions between the amine hydrogen atoms and the nearby located ring hydrogen atoms in determining the relative energy of the two tautomers.

The harmonic oscillator model of aromaticity (HOMA) index [18,19,20],

(1) $$\mathrm{HOMA}=1-\sum_{j}\frac{a_{j}}{n_{j}}\sum_{j}{\left(R_{opt}^{j}-R_{i}^{j}\right)}^{2}$$

was used to estimate the degree of aromaticity of the pyrazole ring in the two tautomers. The values of the *α* parameter and C–C, C–N and N–N optimal bond lengths (*R_opt_*) required to calculate the HOMA index were extracted from [19]. In Equation (1), *n* is the number of bonds in the ring of type *j* (C–C, C–N and N–N), and *R_i_* corresponds to the actual bond lengths. The calculated HOMA index for 3AP and 5AP was found to be large, in consonance with the aromatic character of the pyrazole ring and approximately equal (0.87 and 0.88, respectively), demonstrating that the degree of aromaticity of the pyrazole ring in the two tautomers is similar, and it is not a crucial factor in determining their relative energies.

### 2.2. Infrared Spectra of Matrix-Isolated AP

**Figure 2:**
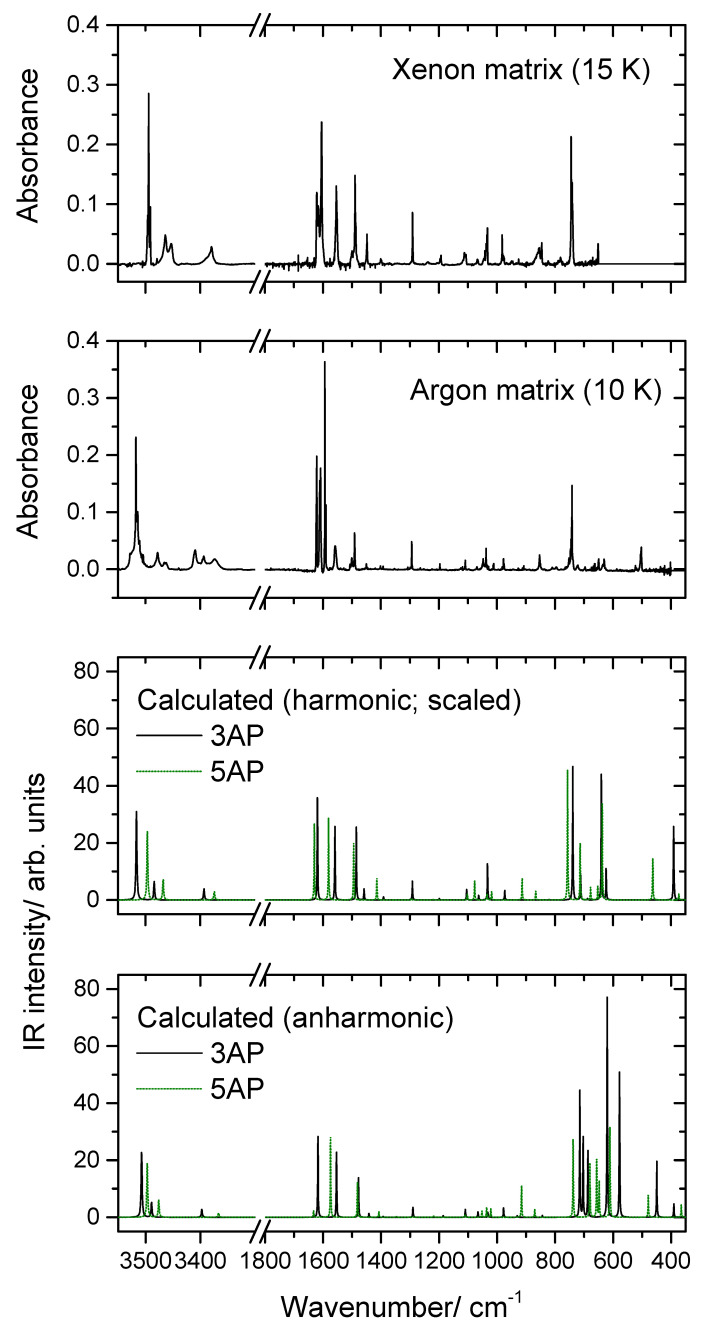
Experimental IR spectra of 3(5)-aminopyrazole isolated in Xe and Ar matrices and B3LYP/6-311++G(d,p) calculated harmonic (scaled) and anharmonic IR spectra for 3AP and 5AP tautomers. Bands due to trace H_2_O impurity were subtracted.

The IR spectra of the matrices prepared using the pyrazole sample (see Section 3.1 for experimental details) are shown in Figure 2. The top two panels in this figure correspond to the mid-IR spectra obtained in xenon and argon matrices, at 15 K and 10 K, respectively, while the two bottom panels of the figure show the DFT(B3LYP)/6-311++G(d,p) computed harmonic (scaled) and anharmonic infrared spectra of 3AP (black solid line) and 5AP (green dotted line). The assignment of the bands to the fundamental modes of the two tautomers is provided in Table 2 and Table 3, respectively, for 3AP and 5AP.

At the first look at the spectra, two main conclusions could be extracted. On one side, the experimental spectra show a general agreement with the calculated spectrum for 3AP (in the region between 500–700 cm^−1^ the anharmonic calculations fail to predict properly the spectra, in particular regarding intensities; this point will be discussed later on), and on the other side, they exhibit a number of extra bands that cannot be ascribed to 3AP, otherwise matching well the predicted frequencies for 5AP. Taken together, these observations indicate that both tautomers are present in the matrices, the lowest energy 3AP tautomer being the dominant species.

The matrices were prepared from a solid sample (labelled as 3AP by the provider) by sublimation, as described in Section 3.1. The crystal structure of the material is not available in the literature, and the characteristics of the solid sample did not allow us to obtain a suitable crystal for X-ray diffraction structure determination. Nevertheless, it seems very much probable that in the solid state the molecules form dimeric units with a pseudo six-membered ring bound by two intermolecular hydrogen bonds, N–H^…^N’ and N’–H’^…^N (where the plica is used to distinguish molecule 1 from molecule 2 in the dimer). Such structural arrangement allows that both tautomers (3AP and 5AP) form upon sublimation, most probably in the solid–gas interface, thus justifying the observation of both tautomers in the matrix. This mechanism is consistent with the dominance of the 3AP tautomer in the studied matrices (and in the gas phase from where the molecules were trapped into the cryogenic media) since its higher stability can be expected to favor its release from the solid to the gas phase in larger amounts. Note that the tautomerization cannot take place in the gas phase because the energy barrier for interconversion between the 3AP and the 5AP tautomers via intramolecular H-shift (~200 kJ mol^−1^) is prohibitive, and no equilibration between the tautomers is expected to take place in the gas phase at room temperature by this mechanism. The relative population of the two tautomers in the gas phase is, otherwise, determined by the chemistry taking place at the solid–gas interface during sublimation. It is also interesting to note that previous studies [16] have revealed that pyrazoles bearing π-electron donating substituents at positions 3 or 5 of the ring, like the 3(5)-aminopyrazoles, may undergo easy double intermolecular proton transfer (as well as solvent-assisted proton transfer), which is a conclusion in line with the explanation given above for observation of both tautomers in the studied matrices.

Considering the general good reproduction of the experimental spectra by the theoretical calculations, the assignment of the spectra was straightforward. Moreover, the assignment of the bands to the tautomers was facilitated by the fact that, as described in the next section, upon UV-irradiation tautomer 3AP converts to tautomer 5AP. There are, however, a few observations that shall be here discussed in more detail:

(i) The first point to note is the fact that several bands appear site split, indicating the existence of multiple trapping sites in both matrices. This is a common feature for matrix isolation IR spectra, and as usual, the bands of higher intensity are those exhibiting more extensive splitting due to the fact that they correspond to more polarized oscillators, which are more sensitive to changes in the local environment.
(ii) As already mentioned, somehow surprisingly the anharmonic calculations fail to reproduce properly the observed spectra in the region between 700 and 500 cm^−1^. The frequencies are also not well predicted, but the estimation of the intensities is very much in error, being by far more intense than the observed ones. Interestingly, the agreement between the calculated harmonic (scaled) frequencies and intensities and the observed spectra, in this spectral region, is much better, describing fairly well the observations. The reason(s) for the failure of the anharmonic calculations in predicting appropriately this spectral region must be scrutinized in a dedicated investigation where several molecules have to be considered, but this lays outside the scope of the present study. Nevertheless, the problem seems to be related to the description of the amine moiety (or, at least, an amine fragment directly bound to an aromatic ring) since most of the vibrations giving rise to bands in this spectral region have major contributions from modes of this molecular fragment, in particular the γ(NH_2_) rocking mode (in line with this reasoning, a strong underestimation of the intensity of the δ(NH_2_) vibration of 5AP by the anharmonic calculations shall also be noticed; see Table 3).
(iii) Among all the calculated frequencies, those obtained for g(NH) mode are the ones predicted as more deviated from the experimental frequencies. In this case, the anharmonic calculations provide considerably better results than the harmonic ones (even after scaling these latter using the same scale factor as for all other vibrations absorbing below 1800 cm^−1^) but still underestimate the observed frequencies by approximately 10% (~50 cm^−1^). In this case, however, one can attribute the disagreement as being mostly determined by interactions between the N–H fragment and the matrix host that make the movement of the hydrogen atom out of the plane of the ring more difficult (i.e., they lead to a larger force constant for this oscillator in the matrix than in the free molecule). Two different (possibly acting simultaneously) types of interactions can be considered: on the one side, packing-related interactions, which can be expected to favor the planarity of the molecule (hence increasing the energy of the system upon the hydrogen atom movement to out of the plane of the ring), and, on the other side, H-bond like interactions, where the N–H group appears as donor and the host matrix atoms as acceptors (this type of interaction is well-known for molecules bearing an O–H moiety [21,22,23] but can also be expected to occur in the case of an N–H group).
(iv) A final note shall be made regarding the origin of the band observed in the argon matrix at 853 cm^−1^. This band can be partially due to the γ(CH)_a_ mode of 3AP (calculated intensity ~1 km mol^−1^; see Table 2), but the observed intensity appears to be too large for this to be the sole contribution to the intensity of the band. It can be tentatively suggested that the intense (~6 km mol^−1^) combination mode predicted by the anharmonic calculations at 833 cm^−1^ also contributes to the observed band. This combination mode is associated with the vibration whose fundamental transition gives rise to the intense band observed at 649 cm^−1^ (a mixed vibration with major contributions from γ(NH_2_) and a torsion of the ring) plus the torsion of the amine group (τ(NH_2_, whose fundamental was predicted at 223 cm^−1^ by the anharmonic calculations).

### 2.3. UV-Induced Phototautomerization

**Figure 3:**
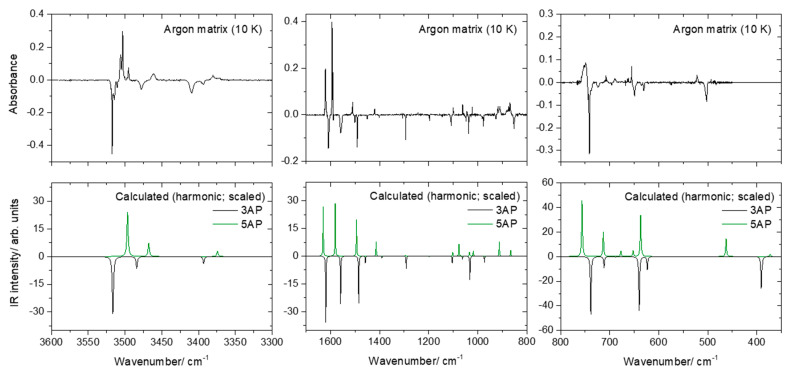
Experimental IR difference spectra obtained by subtracting the spectrum of the as-deposited argon matrix from that obtained after UV irradiation (λ > 235 nm; 5.5 h; 10 K) in different spectral regions (top panels) and DFT(B3LYP)/6-311++G(d,p) calculated IR spectra of 3AP (black) and 5AP (green) calculated within the harmonic approximation. In the calculated spectra, wavenumbers were scaled, and the intensities in the spectrum of 3AP were multiplied by −1.

The deposited argon matrix of 3(5)-aminopyrazole was subjected to in situ irradiation with broadband UV-Vis light (λ > 235 nm), as described in Section 3.1. Figure 3 shows the resulting IR difference spectrum obtained by subtracting the spectrum of the as-deposited matrix from that recorded after irradiation (5.5 h; 10 K). This figure also shows the simulated difference spectrum built based on the calculated harmonic (scaled) IR spectra of 3AP and 5AP.

It is clear from Figure 3 that irradiation induces conversion of tautomer 3AP into 5AP. Minor bands belonging to additional photoproducts were also observed in the IR spectra obtained after irradiation, in particular in the 2240–2260 and 2070–2180 cm^−1^, which with all probability belong to products resulting from the opening of the pyrazole ring. Possible photoproducts include cyanamide (N≡C–NH_2_), ethynamine (H–C≡C–NH_2_), carbodiimide (HN=C=NH) and ketenimine (CH_2_=C=NH), which have the most intense bands in these spectral ranges (and weak bands both above and below these regions that could not be identified in the spectra of the photolysed matrix) [24,25,26]. In any case, the photolysis of the compounds occurs in a reduced extension, leading to very small amounts of the corresponding products, the 3AP → 5AP phototautomerization process being clearly dominant.

Since the molecules are isolated, the observed phototautomerization must occur via intramolecular H-shift. Excitation was performed at λ > 235 nm, which, according to the performed TD-DFT(B3LYP)/6-311++G(d,p) calculations on the most stable 3AP tautomer (see Table S1 and Figure S1, provided as Supplementary Materials), corresponds to excitation within the long-wave wing of the S_3_ ← S_0_ transition, whose maximum was predicted at 223.6 nm (535.1 kJ mol^−1^) with an oscillator strength of 0.0354. The calculations also predict that S_1_ stays 257.5 nm (464.6 kJ mol^−1^) higher in energy than the ground state. This energy is much higher than the calculated energy barrier for the intramolecular H-shift in the ground state (194.4 kJ mol^−1^; for structure of the transition state see Supplementary Materials Figure S2 and Table S2), so that energy relaxation from S_1_ to S_0_ occurs with enough energy being released to overcome the barrier, suggesting that the H-shift occurs, most probably, in the vibrationally excited ground state. Nevertheless, the possibility of the H-shift taking place in the excited state cannot be ruled out on the grounds of the present study and requires further examination through detailed calculations of the excited states’ potential energy surfaces of the studied system.

## 3. Materials and Methods

### 3.1. Experimental Details

A low melting solid sample (m.p. 34–37 °C) labelled as 3-aminopyrazole (99% purity) was purchased from Fluorochem (Hadfield, Glossop, UK), with spectroscopic grade. Prior to usage, the sample was placed in a glass tube and connected to the vacuum chamber of a helium-cooled cryostat through a needle valve. Matrices were prepared by co-deposition of vapors of the compound together with a large excess of the matrix host-gas (Ar N60, or Xe N48), obtained from Air Liquide] onto a CsI substrate assembled at the cold (10 K in argon experiments; 15 K in xenon experiments) tip of the cryostat (APD Cryogenics closed-cycle helium refrigeration system with a DE-202A expander in the case of the argon matrices, or ARS Cryogenics closed-cycle helium refrigeration system with a DE-202SI expander for the xenon matrices). The temperature of the CsI window was measured directly at the sample holder by a silicon diode sensor connected to a digital temperature controller (Scientific Instruments, model 9650−1 or LakeShore 335), which provides an accuracy of ±0.1 K. Both the valve nozzle and the sample glass container were kept at room temperature (298 K).

The IR spectra were obtained using a Thermo Nicolet 6700 Fourier (Thermo Electron Corporation, Waltham, MA, USA) transform infrared spectrometer, equipped with a deuterated triglycine sulfate (DTGS) detector and a Ge/KBr beam splitter for the studies performed on the argon matrices, or a Thermo Nicolet iS50 Fourier transform infrared spectrometer equipped with an MCT/A detector and a KBr beam splitter for the studies undertaken on the xenon matrices. The spectral ranges covered were 400–4000 cm^−1^ and 650–4000 cm^−1^ for argon and xenon experiments, respectively, the spectral resolution being 0.5 cm^−1^ for both cases. To avoid interference from atmospheric H_2_O and CO_2_, a stream of dry and CO_2_-filtered air was continuously purging the optical path of the spectrometers.

Broadband irradiation of the matrices was carried out with UV light provided by a 500 W high-pressure Hg(Xe) lamp (Oriel Instruments, Newport, RI, USA), with output power set to 250 W, through the outer KBr window of the cryostat (λ > 235 nm, as defined by the onset of KBr transmission in the UV).

### 3.2. Computational Details

The quantum chemical calculations were performed using the Gaussian 16 program package (Rev. B.01) [27] at the DFT(B3LYP) [28,29,30] level of theory using the 6-311++G(d,p) basis set [31,32]. All geometries were optimized using the TIGHT convergence criteria of Gaussian 16, and the nature of all described stationary points was further characterized through the analysis of the corresponding Hessian matrices. Transition state structures were located using the synchronous transit-guided quasi-Newton (STQN) method [33]. The UV absorption spectra were computed at the same level, using the same functional and basis set [34,35]. Vibrational calculations were performed both using harmonic and anharmonic potentials, and in the estimation of relative energies, the zero-point vibrational energy (ZPVE) corrections were also accounted for. Harmonic wavenumbers were scaled by 0.955 and 0.980, respectively, above and below 3000 cm^−1^. Anharmonic IR spectra were computed using the fully automated second-order vibrational perturbative approach developed by Barone and co-workers [36,37], allowing for the evaluation of anharmonic infrared intensities up to 2 quanta, including overtones and combination bands [37,38,39]. The resulting wavenumbers together with the calculated IR intensities were used to simulate the spectra shown in the figures through convolution with Lorentzian functions having a full-width-at-half-maximum (fwhm) equal to 2 cm^−1^. The vibrational analysis was supported by the animation of the vibrations of both tautomers provided by Chemcraft software [40].

## 4. Conclusions

In the present research, 3(5)-aminopyrazole was investigated in argon and xenon matrices and by quantum chemical calculations at the DFT(B3LYP)/6-311++G(d,p) level of theory. The structure of the two tautomers, 3AP and 5AP, was compared and the reasons for their relative energy evaluated. It was found that both electronic interactions involving the lone-electron pair of the amine group nitrogen atom and the π-system of the pyrazole ring as well as electrostatic repulsions between the amine nitrogen atoms and the closest located ring hydrogen atoms are relevant factors in determining the relative stability of the tautomers, while the degree of aromaticity of the pyrazole ring in the two forms is similar. The assignment of the IR spectra obtained in both studied matrices was undertaken, revealing the presence in the matrices of the two tautomers, 3AP and 5AP, and a mechanism was postulated to explain the simultaneous presence of the two tautomeric species in the as-deposited matrices. Interpretation of the spectra was supported by vibrational calculations carried out at both harmonic and anharmonic levels, and the abilities of the two types of calculations as applied to the present case study were discussed.

The matrix-isolated compound (in argon matrix) was subjected to in situ broadband UV irradiation (λ > 235 nm), and the UV-induced transformations were followed by IR spectroscopy, revealing that, under the used experimental conditions, phototautomerization of the 3AP tautomer into the 5AP form strongly predominates over pyrazole ring opening photolysis. According to the performed TD-DFT calculations, the bright-state in the photochemical experiments corresponds to S_3_ (of the 3AP tautomer), which upon decay may lead to a vibrationally excited S_0_ state that has enough energy to surpass the energy barrier for H-shift, resulting in the observed tautomeric process.

## Figures and Tables

**Table 1:** B3LYP(6-311++G(d,p) calculated selected relevant structural parameters for 3AP and 5AP *^a^*.

Parameter	3AP	Parameter	5AP
C_3_–N_8_	1.395	C_5_–N_8_	1.399
C_3_–N_2_	1.329	C_3_–N_2_	1.326
C_5_–N_1_	1.353	C_5_–N_1_	1.359
N_1_–N_2_	1.359	N_1_–N_2_	1.360
C_3_–C_4_	1.421	C_3_–C_4_	1.415
C_4_–C_5_	1.379	C_4_–C_5_	1.384
N_1_–H_6_	1.006	N_1_–H_6_	1.007
N_8_–H_9_	1.012	N_8_–H_9_	1.013
N_8_–H_10_	1.010	N_8_–H_10_	1.011
C_3_–N_8_–H_9_	112.6	C_5_–N_8_–H_9_	114.1
C_3_–N_8_–H_10_	114.2	C_5_–N_8_–H_10_	112.4
H_9_–N_8_–H_10_	111.5	H_9_–N_8_–H_10_	109.9
N_2_–C_3_–N_8_–H_9_	15.1	N_1_–C_5_–N_8_–H_9_	59.2
N_2_–C_3_–N_8_–H_10_	143.6	N_1_–C_5_–N_8_–H_10_	−174.8

*^a^* Bond lengths in Å; bond angles in degrees. For atom numbering, see Figure 1.

**Table 2:** Experimental IR bands assigned to 3AP isolated in Ar and Xe matrices and DFT(B3LYP)/6-311++G(d,p) calculated IR data for 3AP, with tentative assignments *^a^*.

	Experimental		Calculated (Harmonic)		Calculated (Anharmonic)	
Approximate Description	Ar Matrix	Xe Matrix	ν	I^IR^	ν	I^IR^
ν(NH)	3519.0	3496.0	3516.4	97.6	3507.3	73.9
	3517.5	3494.0				
	3515.5	3491.0				
	3514.0					
	3511.5					
	3510.5					
ν(NH_2_)_a_	3478.0	3464.5	3484.1	20.4	3489.1	16.4
ν(NH_2_)_s_	3409.0	3379.5	3393.1	12.3	3397.4	8.6
	3393.5					
ν(CH)_s_	3182.5	3181.0 (?)	3114.6	0.4	3137.5	1.4
ν(CH)_a_	3151.0	3157.0	3097.7	1.4	3118.2	2.3
δ(NH_2_)	1611.0	1604.5	1618.8	118.9	1617.6	90.6
ν(C4C5) − ν(C3N2)	1557.0	1554.0	1558.5	80.8	1553.5	71.8
ν(C3C4) − ν(C3N8)	1500.5	1489.0	1484.8	82.1	1477.7	44.5
	1491.0	1486.5				
δ(NH) + ν(C5N1)	1450.0	1448.0	1458.0	12.1	1441.7	4.3
ν(C4C5) + ν(C3N2) − ν(C3C4)	1401.5	1400.0	1390.9	3.5	1392.5	0.8
δ(C5H) + δ(C4H) + δ(NH)	1294.0	1291.0	1291.4	20.9	1290.0	11.0
ν(NN)	1196.5	1195.0	1198.6	1.5	1185.9	1.7
		1193.0				
tw(NH_2_) + ν(C5N1)	1109.0	1112.0	1104.2	11.9	1109.4	8.5
		1106.0				
δ(C4H) + tw(NH_2_)	1048.5	1068.0	1062.8	5.1	1065.9	6.0
δ(C5H) + ν(NN)	1037.5	1041.5	1032.4	40.1	1031.8	5.6
		1036.5				
		1034.5				
		1032.5				
ν(C3C4) + δ(ring-NNC3)	982.0	982.0	973.1	10.4	977.7	10.4
	977.0	978.0				
	976.0	975.0				
δ(ring-NNC5)	927.0	925.0	922.0	0.1	930.7	1.7
γ(CH)_a_	853.0(?)	n.obs.	820.3	0.4	843.9	1.8
γ(CH)_s_ + γ(NH_2_) + τ(ring)	742.0	741.0	738.6	147.4	715.0	139.3
	741.0	739.0				
γ(CH)s − γ(NH_2_)	723.0	718.0	711.5	29.2	703.5	87.6
ν(C3N8) + δ(ring-CCN2)	696.0	694.5	687.8	1.3	686.9	74.7
γ(NH_2_) + τ(ring)	649.0	651.0	639.9	140.0	620.4	245.1
τ(ring) + γ(NH)	635.0	n.i.	623.4	34.5	578.1	160.4
	630.5					
γ(NH)	503.1	n.i.	391.0	79.6	449.5	61.6
δ(C3N8)	n.i.	n.i.	388.6	9.2	390.6	15.0
γ(C3N8)	n.i.	n.i.	299.8	17.0	328.7	28.1
τ(NH_2_)	n.i.	n.i.	241.0	39.3	223.2	36.0

*^a^* Wavenumbers (ν) in cm^−1^; calculated intensities (I^IR^) in km mol^−1^; s = symmetric; a = antisymmetric; ν = stretching; δ = in-plane bending; γ = out-of-plane rocking; tw = twisting; τ = torsion; n.obs. = not observed; n.i. = not investigated: ? = uncertain.

**Table 3:** Experimental IR bands assigned to 5AP isolated in Ar and Xe matrices and DFT(B3LYP)/6-311++G(d,p) calculated IR data for 5AP, with tentative assignments *^a^*.

	Experimental		Calculated (Harmonic)		Calculated (Anharmonic)	
Approximate Description	Ar Matrix	Xe Matrix	ν	I^IR^	ν	I^IR^
ν(NH)	3505.0	3485.0	3496.6	76.0	3497.1	59.6
	3503.0	3479.0				
	3495.5					
ν(NH_2_)_a_	3461.0	3452.0	3467.7	23.7	3476.1	19.0
ν(NH_2_)_s_	3380.0	3358.0	3374.3	9.4	3366.9	4.9
	3371.5					
ν(CH)_s_	3163.0	3176.0 (?)	3106.7	1.4	3129.6	2.0
ν(CH)_a_	3145.0	3146.0	3083.3	6.8	3103.4	8.3
δ(NH_2_)	1620.5	1615.5	1629.6	84.5	1632.7	7.3
ν(C4C5) − ν(C5N8)	1593.5	1586.5	1580.4	90.3	1574.1	88.8
	1589.5					
ν(C5N1) + ν(C3N2)	1510.5	1499.5	1493.8	64.9	1481.3	40.0
δ(NH) + ν(C5N1)	1420.0	1401.0	1414.1	24.2	1407.6	6.4
ν(C3N2) + ν(C3C4)+δ(C4H)	1353.0 (?)	n.obs.	1352.5	0.6	1346.9	0.8
δ(C3H) + δ(NH)	n.obs.	n.obs.	1294.2	1.7	1289.4	0.6
δ(C3H) + δ(C4H)	1243.0 (?)	1242.0 (?)	1219.5	0.7	1219.0	1.4
tw(NH_2_) + ν(C5N1)	1100.5	n.obs.	1102.2	6.6	1110.7	2.4
ν(NN) + τω(NH_2_)	1063.0	1057.5	1076.9	20.7	1052.4	7.2
δ(C3H)–δ(C4H)+ν(C3C4)	1045.0	n.obs.	1034.2	7.2	1036.2	11.3
	1041.0					
ν(C4C5) + ν(C5N1)	1023.5	1006.0 (?)	1018.4	9.2	1021.8	9.4
δ(ring-NNC3)	919.0	898.5	913.0	24.1	915.6	35.5
	917.0					
	910.0					
γ(C3H)	888.5	855.0	886.3	9.9	870.2	8.6
	881.5	845.0				
	875.0					
	872.0					
	869.0					
	867.5					
γ(C4H)	752.2	730.0	756.4	143.6	737.9	86.9
γ(NH_2_) − γ(C4H)	707.0	702.0	713.1	62.5	680.3	63.0
δ(ring-CCN1) + ν(C5N8)	690.0	668.0	677.3	14.8	656.7	66.7
τ(ring)	663.5	660.0 (?)	652.3	15.5	648.0	39.6
t(ring) + γ(NH)	655.5	655.0 (?)	636.9	106.8	611.0	99.1
γ(NH)	523.0	n.i.	462.8	47.9	478.8	25.4
δ(CN8)	n.i.	n.i.	373.0	6.6	365.2	14.1
γ(CN8)	n.i.	n.i.	275.9	7.3	277.3	9.5
τ(NH_2_)	n.i.	n.i.	138.2	45.3	125.2	37.1

*^a^* Wavenumbers (ν) in cm^−1^; calculated intensities (I^IR^) in km mol^−1^; s = symmetric; a = antisymmetric; ν = stretching; δ = in-plane bending; γ = out-of-plane rocking; tw = twisting; τ = torsion; n.obs. = not observed; n.i. = not investigated: ? = uncertain.

## Data Availability

Not applicable.

## Supplementary Materials

The following are available online, Table S1: The TD-DFT(B3LYP)/6-311++G(d,p) calculated transition energies (to the 6 lowest singlet excited states) of the 3AP tautomer and oscillator strengths, Table S2: The Cartesian coordinates of the ground state transition state for intramolecular tautomerization as calculated at the DFT(B3LYP)/6-311++G(d,p) level, Figure S1: The simulated TD-DFT(B3LYP)/6-311++G(d,p) UV spectrum of 3AP, and Figure S2: The graphical structure of the DFT(B3LYP)/6-311++G(d,p) calculated ground state transition state for intramolecular tautomerization.

## Sample Availability

Samples of the compounds are not available from the authors.

## References

[1] Karrouchi K., Radi S., Ramli Y., Taoufik J., Mabkhot Y.N., Al-Aizari F.A., Ansar M. (2018). Synthesis and Pharmacological Activities of Pyrazole Derivatives: A Review. Molecules.

[2] Mert S., Kasimogullari R., Ok S. (2014). A Short Review on Pyrazole Derivatives and Their Applications. J. Postdr. Res..

[3] Castillo J.C., Portilla J. (2018). Recent advances in the synthesis of new pyrazole derivatives. Targets Heterocycl. Syst..

[4] Kumari S., Paliwal S., Chauhan R. (2014). Synthesis of Pyrazole Derivatives Possessing Anticancer Activity: Current Status. Synth. Commun..

[5] Anwar H.F., Elnagdi M.H. (2009). Recent developments in aminopyrazole chemistry. Arkivoc.

[6] Fichez J., Busca P., Prestat G. (2017). Recent Advances in Aminopyrazoles Synthesis and Functionalization. Targets Heterocycl. Syst..

[7] Lim F.P.L., Tan K.C., Luna G., Tiekink E.R., Dolzhenko A.V. (2019). A new practical synthesis of 3-amino-substituted 5-aminopyrazoles and their tautomerism. Tetrahedron.

[8] Shaabani A., Nazeri M.T., Afshari R. (2018). 5-Amino-pyrazoles: Potent reagents in organic and medicinal synthesis. Mol. Divers..

[9] Emelina E.E., Petrov A.A., Filyukov D.V. (2014). Structure and tautomerism of 4-substituted 3(5)-aminopyrazoles in solution and in the solid state: NMR study and Ab initio calculations. Russ. J. Org. Chem..

[10] Dorn H. (1973). Tautomerie und Nomenklatur der? Pyrazolone? und Amino-pyrazole. J. Prakt. Chem..

[11] Gonzalez E., Faure R., Vincent E.-J., Espada M., Elguero J. (1979). Etude en Résonance Magnétique Nucléaire du Carbone-13 de Quelques Aminopyrazoles. Sur le Problème de la Détermination des Constantes d’Equilibre Tautomère. Magn. Reson. Chem..

[12] Puello J.Q., Obando B.I., Foces-Foces C., Infantes L., Claramunt R.M., Cabildo P., Jiménez J., Elguero J. (1997). Structure and tautomerism of 3(5)-amino-5(3)-arylpyrazoles in the solid state and in solution: An X-ray and NMR study. Tetrahedron.

[13] Marín-Luna M., Alkorta I., Elguero J. (2015). A theoretical study of the gas phase (proton affinity) and aqueous (pKa) basicity of a series of 150 pyrazoles. New J. Chem..

[14] Catalan J., Menéndez M., Laynez J., Claramunt R.M., Bruix M., De Mendoza J., Elguero J. (1985). Basicity of azoles. VII. Basicity ofC-aminopyrazoles in relation to tautomeric and protonation studies. J. Heterocycl. Chem..

[15] Jarończyk M., Dobrowolski J.C., Mazurek A.P. (2004). Theoretical studies on tautomerism and IR spectra of pyrazole derivatives. J. Mol. Struct. THEOCHEM.

[16] Chermahini A.N., Teimouri A. (2014). Theoretical studies on proton transfer reaction of 3(5)-substituted pyrazoles. J. Chem. Sci..

[17] Secrieru A., O’Neill P.M., Cristiano M.L.S. (2019). Revisiting the Structure and Chemistry of 3(5)-Substituted Pyrazoles. Molecules.

[18] Kruszewski J., Krygowski T. (1972). Definition of aromaticity basing on the harmonic oscillator model. Tetrahedron Lett..

[19] Krygowski T.M., Cyrański M.K. (2001). Structural Aspects of Aromaticity. Chem. Rev..

[20] Cyrański M.K. (2005). Energetic Aspects of Cyclic Pi-Electron Delocalization: Evaluation of the Methods of Estimating Aromatic Stabilization Energies. Chem. Rev..

[21] Ildiz G.O., Nunes C.M., Kuş N., Fausto R. (2012). FTIR investigation of the O–H··Xe interaction in simple carboxylic acids in solid xenon. J. Chem. Phys..

[22] Lopes S., Domanskaya A., Fausto R., Räsänen M., Khriachtchev L. (2010). Formic and acetic acids in a nitrogen matrix: Enhanced stability of the higher-energy conformer. J. Chem. Phys..

[23] Kus N., Sharma A., Peña I., Bermúdez M.C., Cabezas C., Alonso J.L., Fausto R. (2013). Conformers of β-aminoisobutyric acid probed by jet-cooled microwave and matrix isolation infrared spectroscopic techniques. J. Chem. Phys..

[24] Bernstein M.P., Sandford S.A., Allamandola L.J. (1997). The Infrared Spectra of Nitriles and Related Compounds Frozen in Ar and H2O. Astrophys. J..

[25] Bégué D., Qiao G., Wentrup C. (2012). Nitrile Imines: Matrix Isolation, IR Spectra, Structures, and Rearrangement to Carbodiimides. J. Am. Chem. Soc..

[26] Jacox E., Milligan D.E. (1963). Infrared Study of the Reactions of CH_2_ and NH with C_2_H_2_ and C_2_H_4_ in Solid Argon. J. Am. Chem. Soc..

[27] Frisch M.J., Trucks G.W., Schlegel H.B., Scuseria G.E., Robb M.A., Cheeseman J.R., Scalmani G., Barone V., Petersson G.A., Nakatsuji H. (2016). Gaussian 16 Revision B.01.

[28] Vosko S.H., Wilk L., Nusair M. (1980). Accurate spin-dependent electron liquid correlation energies for local spin density calculations: A critical analysis. Can. J. Phys..

[29] Becke A.D. (1988). Density-functional exchange-energy approximation with correct asymptotic behavior. Phys. Rev. A.

[30] Lee C., Yang W., Parr R.G. (1988). Development of the Colle-Salvetti correlation-energy formula into a functional of the electron density. Phys. Rev. B.

[31] McLean A.D., Chandler G.S. (1980). Contracted Gaussian basis sets for molecular calculations. I. Second row atoms, Z = 11–18. J. Chem. Phys..

[32] Krishnan R.S., Binkley J.S., Seeger R., Pople J.A. (1980). Self-consistent molecular orbital methods. XX. A basis set for correlated wave functions. J. Chem. Phys..

[33] Peng C., Schlegel H.B. (1993). Combining Synchronous Transit and Quasi-Newton Methods to Find Transition States. Isr. J. Chem..

[34] Adamo C., Jacquemin D. (2013). The calculations of excited-state properties with Time-Dependent Density Functional Theory. Chem. Soc. Rev..

[35] Laurent A.D., Adamo C., Jacquemin D. (2014). Dye chemistry with time-dependent density functional theory. Phys. Chem. Chem. Phys..

[36] Barone V. (2005). Anharmonic vibrational properties by a fully automated second-order perturbative approach. J. Chem. Phys..

[37] Bloino J., Barone V. (2012). A second-order perturbation theory route to vibrational averages and transition properties of molecules: General formulation and application to infrared and vibrational circular dichroism spectroscopies. J. Chem. Phys..

[38] Barone V., Biczysko M., Bloino J. (2014). Fully anharmonic IR and Raman spectra of medium-size molecular systems: Accuracy and interpretation. Phys. Chem. Chem. Phys..

[39] Barone V., Bloino J., Guido C.A., Lipparini F. (2010). A fully automated implementation of VPT2 Infrared intensities. Chem. Phys. Lett..

[40] (2021). Chemcraft—Graphical Software for Visualization of Quantum Chemistry Computations. https://www.chemcraftprog.com.

