# Supplementary material for: Structure and IR Spectra of 3(5)-Aminopyrazoles and UV-Induced Tautomerization in Argon Matrix
Source: Molecules. 2021 Jul 15;26(14):4299. doi: 10.3390/molecules26144299 (PMC8304260; doi:10.3390/molecules26144299)
Supplement: Supplementary file 1 [file molecules-26-04299-s001.zip › molecules-1295215-supplementary.pdf]

Supplementary Material for

Structure and IR Spectra of 3(5)-Aminopyrazoles and UV-Induced Tautomerization in Argon Matrix

Alina Secrieru [1,2], Susy Lopes [3]*, Maria L. S. Cristiano [2] and Rui Fausto [3]

[1] CCMAR and Department of Chemistry and Pharmacy, FCT, University of Algarve, Campus de Gambelas, 8005-039 Faro, Portugal; asecrieru@ualg.pt (A.S); mcristi@ualg.pt (M.L.S.C)
[2] Department of Chemistry, University of Liverpool, Liverpool L69 7ZD, United Kingdom;
[3] CQC-IMS, Department of Chemistry, University of Coimbra, 3004-535 Coimbra, Portugal; susylopes@qui.uc.pt (S.L); rfausto@ci.uc.pt (R.F)
* Correspondence: susylopes@qui.uc.pt

INDEX

Table S1. TD-DFT(B3LYP)/6-311++G(d,p) calculated transition energies (to the 6 lowest singlet excited states) of the the 3AP tautomer and oscillator strengths.

Figure S1. Simulated UV spectrum of the 3AP tautomer obtained from TD-DFT calculation at the DFT(B3LYP)/6-311++G(d,p) level of theory.

Table S1 – TD-DFT(B3LYP)/6-311++G(d,p) calculated transition energies (to the 6 lowest singlet excited states) of the 3AP tautomer and oscillator strengths.

State	Symmetry	Energy/ nm	Oscillator Strength	Approximate description
S_1	A	257.5	0.0050	HOMO → LUMO
S_2	A	250.2	0.0003	HOMO → LUMO +1
S_3	A	223.6	0.0354	HOMO → LUMO +2
S_4	A	222.0	0.0147	HOMO → LUMO +3
S_5	A	214.3	0.0048	HOMO –1 → LUMO
S_6	A	207.7	0.0024	HOMO → LUMO +4

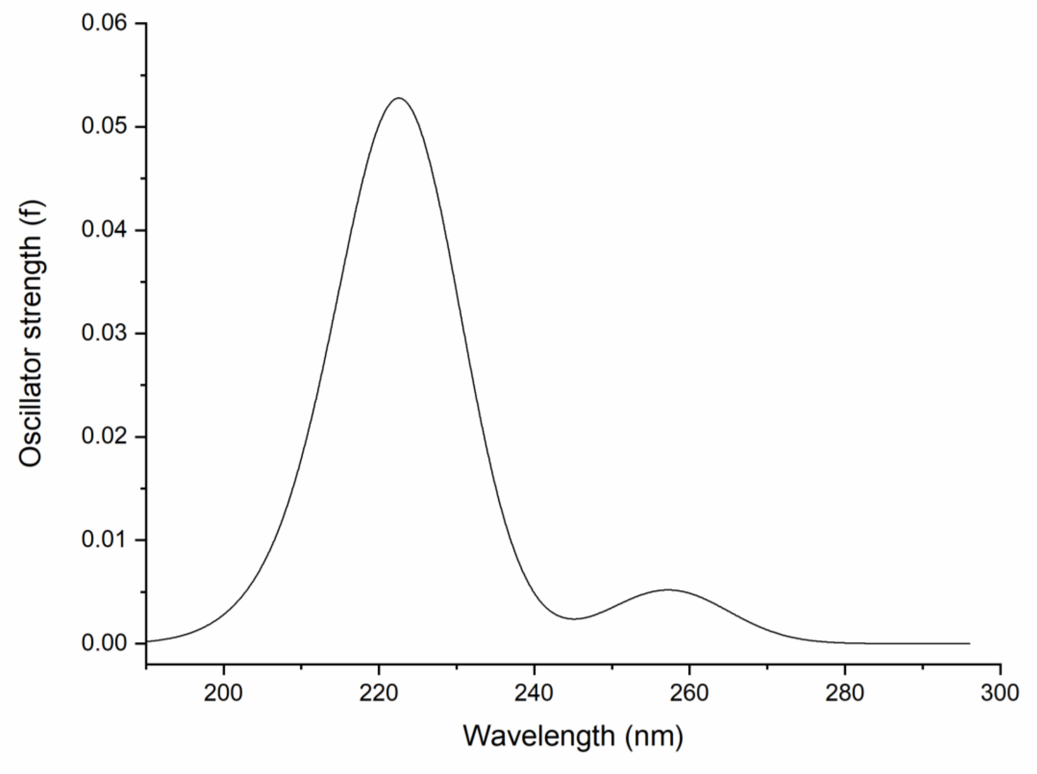

Figure S1. Simulated UV spectrum of the 3AP tautomer obtained from the TD-DFT(B3LYP)/6-311++G(d,p) calculations. A band width at half-height of 17.9 nm was used to simulate the spectrum.
